# Quality of Life and Clinical Impairment in Spanish Adolescent Anorexia Nervosa Patients

**DOI:** 10.3390/ejihpe14050094

**Published:** 2024-05-15

**Authors:** Marie-Carmen Neipp, Álvaro Ruiz, Javier Manchón, Eva León-Zarceño, María José Quiles, Yolanda Quiles

**Affiliations:** 1 Department of Health Psychology, University of Miguel Hernández, 03202 Elche, Spain; 2 Department of Behavioural Sciences and Health, University of Miguel Hernández, 03202 Elche, Spain; alvaro.ruiz03@goumh.umh.es (Á.R.); jmanchon@umh.es (J.M.); eleon@umh.es (E.L.-Z.); mj.quiles@umh.es (M.J.Q.); y.quiles@umh.es (Y.Q.)

**Keywords:** eating disorder behaviors, quality of life, clinical impairment, adolescents

## Abstract

Eating disorders have serious physical, mental and social consequences that can affect the quality of life of the sufferer. This study aimed to evaluate the relationship between the severity of ED-related psychopathology and clinical impairment in adolescents with anorexia nervosa (AN) as well as their perception of health-related quality of life. Eighty-six Spanish young women with AN completed a set of questionnaires assessing eating disorder pathology, clinical impairment, and quality of life. The set included the following instruments: the Eating Disorder Examination Questionnaire, Clinical Impairment Assessment, Short Form-12 Item Health Survey, and the Eating Disorder-Specific Heath-Related Quality of Life instrument. Descriptive and regression analyses were applied to identify associations between variables. Higher scores on clinical impairment domains were associated with greater impairment of mental and physical health. Moreover, clinical impairment domains and concerns due to ED were related to a lower quality of life. In conclusion, adolescents with AN have a poor quality of life. Moreover, the findings suggest that the clinical features of impairment may serve as severity indicators of quality of life.

## 1. Introduction

Eating disorders (EDs) have serious physical, psychological, social, and familial consequences for the people who suffer from them. As stated by Treasure et al. [1], “eating disorders are disabling, deadly, and costly mental disorders that considerably impair physical health and disrupt psychosocial functioning”. Long-term studies have shown considerable rates of persisting ED pathology [2]. This protracted ED pathology means that EDs have a significant impact on the present and future health and quality of life (QoL) of affected individuals, their caregivers, and society [3].

Traditionally recognized for their effects on physical and psychological well-being, EDs have increasingly been associated with impairments across other vital domains of daily life, encompassing employment, education, familial, social, and recreational pursuits [4,5]. Recent investigations have revealed that individuals with EDs exhibit markedly lower QoL and diminished social functioning compared to non-ED counterparts, with psychological well-being often failing to reach the level of healthy controls, even post treatment [2,6,7]. Furthermore, a review of the literature has indicated that individuals with EDs experience a more pronounced decline in QoL than those diagnosed with other psychiatric conditions, including severe depression [4,5,8].

Several studies have attempted to identify the predictors of QoL in these patients and its association with other clinical variables. In the specific case of anorexia nervosa (AN), the literature has shown that AN symptoms and comorbidity are related to more significant QoL impairments [9]; however, the results are heterogeneous. For example, Başoğlu et al. [10] showed that in AN, extreme calorie restriction was associated with several psychological and neurological complications, which can affect these patients’ daily functioning. In this line, Mason et al. [11] showed that dietary restriction was related to lower QoL, and the severity of restriction and bulimic behaviors could serve as severity indicators of QoL in AN. Weigel et al. [12] conducted a study that included both adults and adolescents with AN. Their research showed that, in both age groups, lower BMI, increased levels of depression, and more somatic complaints were associated with lower health-related QoL [12]. However, this research also revealed that neither the duration of the AN nor its psychopathology were associated with QoL [12]. Gonzalez-Pinto et al. [13] indicated an association between psychiatric comorbidity and purging behaviors in these the QoL of those with AN. When the subtype of the AN is considered, Martin et al. [14] showed that patients with restrictive AN experienced a higher QoL than patients with purgative AN.

All the aforementioned points underscore the importance of considering not only symptom improvement but also the impacts of AN on various aspects of patients’ lives, such as their social interactions, family dynamics, and academic or occupational functioning [15]. Understanding which of the symptoms and behaviors of AN are associated with QoL can provide valuable insights into treatment targets. Therefore, this cross-sectional study aims to explore the QoL of adolescent patients with AN, on which studies in the literature are scarce. This study seeks to analyze how factors related to the disorder (ED symptoms, AN subtype, duration of illness, and BMI) are associated with QoL. Additionally, we aim to evaluate these associations using both a generic QoL measurement and a specific ED–QoL scale. It is hypothesized that a lower BMI, increased levels of ED psychopathology, and greater psychological distress will be linked to poorer QoL in adolescent patients with AN.

## 2. Materials and Methods

### 2.1. Participants

Patients (86 female adolescents diagnosed with AN) were recruited between March 2021 and May 2023 from six different specialist outpatient, daycare, and inpatient ED settings in Comunidad Valenciana and Murcia (Spain). A randomized controlled trial study with a longitudinal design was used (see protocol in Quiles et al. [16]). The research assistant at each center conducted a semi-structured interview to evaluate participants and confirm fulfillment of the following inclusion criteria: (1) aged between 11 and 19; (2) AN diagnosis according to the fifth edition of the Diagnostic and Statistical Manual of Mental Disorders (DSM-V) criteria [17]; (3) no psychiatric comorbidity; and (4) receiving treatment for AN at a specialist outpatient/day-patient/inpatient ED unit.

### 2.2. Instruments

An ad hoc sociodemographic questionnaire was used to assess age and educational level.

An ad hoc clinical variables questionnaire was submitted by the health care providers at the unit of reference. It assessed diagnosis (according to DSM-V criteria), weight, BMI, and AN time-course.

The Eating Disorder Examination Questionnaire (EDEQ) [18] was used to assess the severity of psychopathology related to eating disorders. It comprised 36 items rated on a six-point Likert scale, divided into four dimensions: Restraint, Eating Concern, Shape Concern, and Weight Concern. Higher scores indicate greater severity of ED features. The cut-off point was ≥20 for the total scale and ≥4 for the subscales, indicating clinical significance. The Spanish validation showed satisfactory internal consistency for the dimensions (α = 0.83, α = 0.75, α = 0.93, and α = 0.74, respectively) as well as in the overall scale (α = 0.81) [19].

Clinical Impairment Assessment (CIA 3.0) [20] was used to assess psychological impairment related to the features of ED. CIA 3.0 includes 16 items rated on a four-point Likert scale, divided into three subscales of impairment: Personal, Social, and Cognitive. Higher scores on each scale indicate greater severity of clinical impairment. The cut-off point is ≥16 on the global impairment scale, predicting ED case status. The Spanish validation study showed satisfactory internal consistency with the subscales (α = 0.92, α = 0.93, and α = 0.90, respectively) as well as with the overall scale (α = 0.96) [21]. Despite being originally designed for adult populations, CIA has been previously administered in adolescent and young adult populations [22,23,24]. In this study, it was shown to have satisfactory internal consistency with the subscales (α = 0.93, α = 0.88, and α = 0.83, respectively) as well as with the overall scale (α = 0.94).

Eating Disorders Quality of Life (EDQOL) [6] assesses health-related QoL in ED patients. It comprises 25 items rated on a five-point Likert scale. The instrument is divided into four subscales: Psychological, Physical/Cognitive, Work/School, and Financial. The Spanish validation study showed satisfactory internal consistency with the subscales (α = 0.91, α = 0.85, α = 0.79, and α = 0.79, respectively) and the overall scale (α = 0.91) [25]. Higher scores indicate poorer health-related QoL.

Health Survey (SF-12) [26] was used to measure health-related QoL. It comprises 12 items rated on a three-to-four-point Likert scale, distributed over eight scales: Physical Health (General Health, Physical Functioning, Role Physical, and Body Pain) and Mental Health (Vitality, Social Functioning, Role Emotional, and Mental Health). The Spanish validation showed satisfactory internal consistency for each subscale (α = 0.85 and α = 0.78, respectively) [27]. Higher scores indicate better QoL.

### 2.3. Procedure

The data were gathered during the initial evaluation phase of a research study that examined the effectiveness of a skills-based intervention for patients diagnosed with an ED. The study received approval from both the Ethics and Research Integrity Committee of the University Miguel Hernández of Elche and the participating ED specialized units (Trial Identifier: ISRCTN43554732).

After confirmation for eligibility to participate, the research assistant at each center obtained participants’ and carers’ informed consent. Subsequently, patients filled out a self-administered paper-and-pencil questionnaire; the healthcare providers responsible for each case submitted the patients’ clinical data.

### 2.4. Data Analyses

Descriptive analyses (means, standard deviations, and percentages) were used to explore the data. In addition, normality assumptions were assessed. This revealed that the sample was not normally distributed; therefore, non-parametric tests were used to analyze differences and correlations. Wilcoxon and Kruskal–Wallis tests analyzed the differences between AN subtypes, time-course, and QoL. Spearman’s bivariate correlation coefficient was employed to analyze the associations between the study variables. Regression analyses were performed to assess QoL predictors, using the EDEQ and CIA dimensions as independent variables and the EDQOL and SF-12 scores as dependent variables. The SPSS (Statistical Package for the Social Sciences, Version 28) was used for statistical analysis.

## 3. Results

The sample consisted of 86 female adolescents diagnosed with AN (75 restrictive and 11 purgative). Sociodemographic and clinical descriptive data of the participants are shown in Table 1.

Table 2 presents a comprehensive description of the variables investigated, with their mean values, standard deviations, and the range of minimum and maximum scores observed.

First, the studied time period was grouped into the following ranges: less than one year; between 1 and 2 years; between 3 and 4 years; and more than 4 years. A Kruskal–Wallis test was performed, showing no significant differences in any of the variables after the data were divided into the previously mentioned timeframes.

A non-parametric Wilcoxon test was carried out to assess differences between AN subtypes and QoL. Two significant differences were found between the variables: adolescents with purgative AN had worse mental health (*W* = 219.50, *p* = 0.021) and scored higher in the psychological EDQOL dimension (*W* = 214.50, *p* = 0.021) than adolescents with restrictive AN.

Spearman’s correlation analyses were conducted to study the relationship between all the variables (Table 3). Timeframe, age, weight, and BMI did not show significant correlations with any study variables. All dimensions of the EDEQ and CIA showed significant relationships with the QoL variables, all scoring above 0.40 except for the EDQOL Work/School and the SF-12 Physical Health dimensions, which were below 0.40. The Psychological dimension of the EDQOL showed the highest correlations with the EDEQ Eating (*r* = 0.75, *p* < 0.01) and Shape (*r* = 0.75, *p* < 0.01) Concern dimensions, and with the CIA Personal Impairment dimension (*r* = 0.79, *p* < 0.01).

To clarify the factors influencing QoL, we conducted a regression analysis, as shown in Table 4. This analysis utilized independent variables from two sources. The Eating Disorders Exploration Questionnaire (EDEQ) covered the following four dimensions: restraint; eating behaviors; weight concern; and body shape concern. The Clinical Assessment of Impairment (CIA) evaluated the following three dimensions: personal; social; and cognitive impairments. The dependent variables were selected from the Eating Disorders Quality of Life (EDQOL) instrument, which comprises the following four dimensions: psychological; physical/cognitive; economic; and work/school. Two dimensions of the SF-12 health survey, physical and mental health, were also included. No problems of multicollinearity, characterized by high intercorrelations among variables, were identified within any of the regression models.

Concerning the four EDQOL dimensions, higher scores on EDEQ Eating Concern and the CIA Personal Impairment dimensions were related to worse scores on the EDQOL Psychological dimension, explaining 61% of its variance. Two CIA dimensions (Cognitive and Social Impairment) were entered into the model for the EDQOL Physical/Cognitive dimension. These dimensions were found to be predictors of a lower EDQOL Physical/Cognitive score, accounting for 71% of the variance observed in this measure. The EDEQ Eating Concern and the CIA Cognitive and Personal Impairment dimensions explained 32% of the EDQOL Work/School dimension’s variance: higher Cognitive Impairment scores were related to worse Work/School dimension scores. Eating Concern and Personal Impairment were also negatively related to this EDQOL dimension. No predictors were observed for the EDQOL Financial dimension.

Regarding the SF-12 QoL questionnaire, higher scores on the EDEQOL Restraint and the CIA Cognitive Impairment dimensions were related to worse SF-12 Physical Health scores, explaining 31% of its variance. Concerning the SF-12 Mental Health dimension, two CIA dimensions (Social and Cognitive Impairment) were found to have a negative correlation, which explained 61% of the variance in the SF-12 Mental Health dimension.

## 4. Discussion

The study thoroughly examines various factors influencing the QoL among adolescents with AN. Through a comprehensive analysis, the research explores the impact of different variables, including ED psychopathology, various demographic factors, and the dimensions related to ED and impairment. The findings provide valuable insights into the complexities of AN and its repercussions on the QoL of affected individuals.

The results of our study are similar to those found in previous studies in the literature that confirm the existence of strong relationships between suffering from an ED and the deterioration of QoL in adolescent patients [28,29,30]. Studies in the literature have already highlighted the need to increase the number of studies that relate QoL and ED in adolescent populations due to their high risk of suffering from an ED [31]. It is essential to better understand and identify the specific differences in patients’ QoL in different diagnostic groups [32]. The present study was conducted with adolescent patients suffering specifically from AN, which allowed us to delve deeper into the impact of AN on the QoL of this group of patients. Other similar studies have found that patients with AN have a lower psychological and physical/cognitive QoL compared to patients with a BN or an ED not otherwise specified (EDNOS) [33]. Studies have found that patients with purging anorexia had the poorest perception of QoL in all areas assessed [34].

Differences were also detected between the AN subtype and the relationship with adolescents’ QoL, showing that adolescents suffering from purgative AN have worse mental health. Likewise, similar studies showed that patients with restrictive AN had a significantly better QoL than those with purgative AN [35], and that patients with purgative AN had greater psychosocial impairment than patients with restrictive AN [36].

One notable finding is the lack of significant differences for all variables studied based on the different timeframes. This suggests that the duration of the disorder does not necessarily correlate with variations in the measured variables, challenging the assumption that longer durations inherently lead to more severe outcomes, in line with previous studies in the literature [15]. Additionally, no significant correlations were found between timeframe, age, weight, BMI, and the variables under consideration, indicating that these factors may not be reliable indicators of the severity or impact of AN in this population. These results contradict other studies that have found BMI to be the strongest predictor of disease recovery [37]. Therefore, further investigation of the impact of these demographic and clinical factors on the QoL of AN patients should be carried out.

Concerning the objective of analyzing the relationships between ED-related psychopathology factors and AN patients’ QoL, the results yielded significant and high associations between all dimensions of the EDEQ and CIA with QoL variables, except for the EDQOL Work/School and SF-12 Physical Health dimensions, which were low.

In addition, the psychological dimension of the EDQOL questionnaire showed the highest correlations with the EDEQ dimensions of Eating and Shape Concern and also with the Personal Impairment dimension. These findings highlight the intricate interplay between psychological well-being and the severity of eating and shape concerns, suggesting that addressing these aspects is crucial for improving overall QoL in adolescents with AN. Studies indicate that, in AN patients, comorbidity and symptomatology are related to greater QoL impairment, and that these patients are more likely to report bodily pain, depression, self-harming behaviors, and suicidal ideation [9]. Recently, studies have also revealed strong relationships between QoL and depressive symptoms in patients with AN [38]. Therefore, therapeutically, it would be interesting to adjust the specific goals of the intervention based on the patient’s level of physical impairment. This should also be considered when designing intervention programs, adjusting them to the patients’ needs to improve their QoL in different areas, as this is a key factor in their recovery [39].

The strong relationships between attitudes towards eating disorders, clinical impairment, and mental health-related QoL of adolescent girls were analyzed in this study. Thus, attitudes towards eating disorders, clinical impairment, and mental health-related QoL in adolescent girls indicated strong negative relationships between the mental health component of the SF-12 with all dimensions of the EDEQ, EDQOL and CIA. Previous studies in the literature have already highlighted how patients with an eating disorder have more impaired mental health than physical health [14], as well as lower QoL than the general population [40]. Weight concerns and their relationship with clinical deterioration translate into lower QoL, even in pre-adolescents [41]. Therefore, mental health interventions for adolescent patients with ED should also address the psychosocial areas impacted [42], and in areas such as academic studies. In addition, our results have shown strong relationships between social and cognitive impairment and the impact of the eating disorder on labor or academic performance in adolescent girls assessed with the EDQOL. Additionally, studies conducted with adult AN patients have also suggested that patients spend more time at home or alone due to their impaired QoL; this impacts their work or studies and may cause more binge eating leading to a worse QoL [11]. Other authors have highlighted work/study impairment as the strongest predictor of overall QoL impairment in EDs [43]. Our work has found that all EDQOL subscales, with the exception of the Financial dimension, had significant correlations with the EDE scales, which is identical to the results obtained by Mitchison et al. [44]. However, in our study, the two components of the SF-12, physical and mental, obtained correlations with the EDE dimension. In other studies, there has only been a relationship between the EDE and the mental components of the SF-12 [44].

Regarding the second objective, which was to evaluate the different relationships of these variables with the QoL measured from a generic measure (SF-12) and with another specific scale of ED QoL (EDQOL), the results showed that 70% of the variance in physical and cognitive QoL evaluated with the EDQOL scale was explained by the CIA social and cognitive dimensions. A comparison with the mental QoL assessment obtained with a general QoL scale (SF-12), indicated that the results were similar, such that the CIA personal and cognitive dimensions explained 61% of the variance, excluding eating symptomatology variables from the explanatory model. Patients with greater ED psychopathology experienced more significant impairment secondary to ED, as previously reported in other studies using clinical samples [14,18,37,38]. These results are in line with previous studies showing that the QoL of patients with AN is greatly affected by eating symptoms, and that this deterioration increases as the symptoms become more intense [39]. These findings underscore the importance of considering the impact beyond clinical symptoms. Individuals with EDs not only face challenges related to eating and weight but also experience a significant burden in their psychosocial functioning, resulting in a lower QoL. This finding underscores the need to address well-being in all areas of life for adolescents with ED. Therefore, the use of specific instruments and adapted measures for this type of patient, in order to assess their functioning in different areas and in their psychosocial impairment as it relates to QoL, should be a future aim [22].

Moreover, as expected, the EDEQ Eating Concern and CIA Personal Impairment dimensions explained a high percentage of variance (61%) in the psychological dimension of the EDQOL. The AN core symptom, eating concern, is related to higher eating pathology, emotional distress, and psychosocial deterioration in the sense that the more intense the eating symptoms, emotional distress, and psychosocial deterioration, the worse the QoL. This result has been found in previous studies [45,46].

Personal and cognitive impairment are also included as explanatory variables in the case of school and work QoL, together with eating concern. In this case, the explained variance was lower (32%), again highlighting the impairment caused by the core symptoms in patients with AN. On the other hand, the psychological dimension of QoL was explained (61%) by restriction concern and personal impairment. In this case, a core symptom of ED (eating concern) is related to mood and self-perception. Studies with a larger sample should be conducted to assess how this deterioration is associated with the AN time-course, as well as to assess possible differences with other types of ED.

It is remarkable that the cognitive dimension of psychosocial impairment exhibits a noteworthy impact on the physical, school, and mental dimensions of QoL. This confirms that cognitive challenges, such as fixation on body image and self-esteem, exert a profound influence on the lives of adolescents with EDs [47]. These outcomes strongly advocate for implementing interventions specifically designed to address these nuanced aspects within the framework of ED treatment.

Finally, we highlight the fact that neither impairment nor deterioration explained the financial dimension of QoL. This could be because economic concerns are not a central aspect of ED, especially in adolescent girls, and that psychosocial impairment manifests more intensely in other domains of patients’ lives. We think that, due to the age of the patients, who are minors and financially dependent on their families, they do not perceive an impairment in this dimension of their EDQOL. However, this aspect can be significantly altered in their families or in adult patients, for whom the illness can involve a high economic cost and a significant loss of QoL for those affected [29].

Based on the results obtained, we can confirm that the specific QoL instrument (EDQOL) is able to explain a higher percentage of AN in psychological and physical dimensions, while the general questionnaire, SF-32, explains a high percentage of variance in the mental domain but not in the physical domain. Previous studies have highlighted this limitation of the generic tool, SF-32, by pointing out that the increased physical activity associated with improved QoL in the SF-36 may be a sign of severity in anorexia nervosa rather than of improvement [48]. However, in a more recent paper, Panea-Pizarro et al. [49] concluded that the SF-36 could be useful for monitoring the impairment of health in adults ED patients. Therefore, it is necessary to develop studies with a larger number of participants and with a longer follow-up to be able to evaluate the usefulness of these scales for collecting changes throughout treatment.

The present study has some limitations. First, its cross-sectional nature implies that we cannot conclude causality between variables. Further research is needed using a longitudinal design. The typical limitations and advantages of using a self-administered questionnaire must also be considered [50]. As this study was carried out with Spanish participants, its generalizability may be limited due to specific characteristics of the sample, such as demographics, cultural backgrounds, and geographic locations. Results may not be representative of the broader population of adolescents with AN. Another limitation concerns generalization to males. Although the prevalence of ED in females is higher than in males, it is essential to include males to detect possible differences [19]. Future studies should analyze the relationship between QoL and the time progression of ED, and whether the results of this investigation can be transferred to patients with BN or EDNOS. Likewise, given that EDs affect not only the patient but also their entire family and social environment, the impact of support systems on treatment outcomes and QoL should be investigated, and interventions involving and enhancing support from family and friends should be developed. In this report, the CIA 3.0 has been administered in adolescent and young adult populations, as previous studies have done [22,23,24]. Despite showing good reliability scores in this sample, it is worth noting that the CIA is a psychometric tool designed for adult populations. Moreover, the sample size was small because the target population is specific and limited, restricting the availability of subjects for the study. Finally, longitudinal studies should be conducted to explore AN trajectories and how various factors evolve over time. This approach would provide a more dynamic understanding of the disorder, treatment effects, and their implications for QoL.

Among the strengths of this work, we highlight that, to our knowledge, there are few studies to assess the determinants of QoL in adolescent patients with AN. A strength of this study includes the sample we used, which comprises participants with AN from six different ED services. Therefore, it is safe to state that the sample is representative of patients seen in daily clinical practice, and that these results may be generalizable to other populations with ED. Another strength of this study includes the use of a large sample of adolescents with a diagnosis of AN. This is also the first study to examine the QoL of AN patients with a specific QoL questionnaire for adolescent Spanish patients. In addition, there are very limited previous studies that have examined QoL in adolescents with AN; therefore, the current study adds meaningful data to the current literature on the topic.

The results of this study have several practical implications for clinicians, healthcare providers, and researchers working with adolescents with AN. Results provide relevant implications for clinical practice, as they can guide the design of more effective and personalized interventions for patients with AN. By understanding the QoL of these patients, we can address not only the physical symptoms, but also the psychological and social aspects that affect their lives. Previous research revealed that patients receiving treatment can improve their QoL [30]. However, even in the case of remission, patients’ QoL has been found to remain lower than that of the general population [51]. The present study showed that anorexia symptoms were associated with the physical, psychological, and social dimensions of QoL. Cognitive impairment was shown to have a positive and significant relationship with the psychological and social QoL of patients, and a negative relationship with the mental subscale of SF-36. These results make cognitive impairment a relevant target in the treatment of ED. Along the same lines, personal impairment should be the target of intensive therapeutic interventions, as it has been shown to be a psychological and academic determinant. Finally, social impairment was shown to have a significant influence on physical, cognitive, and mental dimensions of QoL. The literature states that core symptoms, such as worries about eating or restriction, are essential targets in the treatment of anorexia. To our knowledge, these results suggest that clinicians should consider the dimensions of QoL as therapeutic goals. In addition, including these measures in assessment protocols can provide viability for the treatment of anorexia. Further implications of the study are as follows: (a) The early detection and timely intervention may help prevent the exacerbation of symptoms and contribute to better long-term outcomes; (b) Given the strong correlations between psychological well-being, eating, and shape concerns with QoL, interventions should specifically address these aspects. Cognitive-behavioral therapy or interventions targeting body image and self-esteem may be beneficial to improve well-being; and (c) Clinicians should conduct comprehensive assessments that consider multiple dimensions, including eating concerns, personal impairment, and cognitive and social impairment. This holistic approach can guide treatment planning in addressing the various facets influencing QoL [52].

## 5. Conclusions

This study showed a deteriorated QoL in adolescent patients suffering from AN. Results showed that patients with a purgative AN diagnosis had worse mental health. Other variables, such as BMI, weight, and timeframe, were not significant. Additionally, the results showed that, although a generic mean QoL is useful for evaluating these patients, it is necessary to develop specific measures, such as EDQOL, that allow for a better understanding of the variability and specificity of these disorders. In summary, this study is one of the only studies to evaluate the QoL of adolescents suffering from ED. Future studies should focus on longitudinal data that allow researchers to observe the changes in QoL in relation to eating symptoms.

## Figures and Tables

**Table 1:** Sociodemographic and clinical descriptive data.

	M	SD	Range	N	%
Age	14.86	1–60	11–19		
Pre-adolescents (11–12 y.o.)				5	5.9
Early adolescents (13–15 y.o.)				52	60.5
Mid adolescents (16–17 y.o.)				26	30.2
Late adolescents (18–19 y.o.)				3	3.5
Education					
Primary school				2	2.3
Secondary school				60	69.8
High school				16	18.6
University				5	5.8
Vocational training				2	2.3
Others				1	1.2
Weight (kg)	41.03	6.92	27–62.4		
BMI	15.93	2.11	11.8–22.30		
AN time-course	20.93	20.83	4–144		
Level of care					
Outpatient setting				24	27.9
Day-patient setting				44	51.2
Inpatient setting				18	20.9

**Table 2:** Descriptive data of variables.

Variables	M	SD	Min.–Max.
EDEQ Restriction	3.44	1.77	0–6
EDEQ Eating concern	3.26	1.36	0–6
EDEQ Shape concern	4.79	1.36	0–6
EDEQ Weight concern	4.11	1.50	0–6
CIA-Personal	12.13	5.01	1–18
CIA-Social	8.60	4.44	0–15
CIA-Cognitive	6.98	3.89	0–15
EDQOL Psychological	3.48	1.15	1–8.5
EDQOL Physical/cognitive	3.10	1.03	1–5
EDQOL Financial	1.14	0.37	1–3.6
EDEQL Work/school	2.46	0.96	1–4.8
SF-12 Physical	12.92	3.10	5–19
SF-12 Mental	14.20	4.11	6–26

Notes: M = Mean; SD = standard deviation; Min = minimum; Max = maximum; EDEQ (Eating Disorders Examination Questionnaire); CIA (Clinical Impairment Assessment); EDQOL (Eating Disorder-Specific Health-Related Quality of Life instrument); SF-12 (Short Form-12 Item Health Survey).

**Table 3:** Spearman’s correlations between eating disorders attitudes, clinical impairment, health-related quality of life.

	1	2	3	4	5	6	7	8	9	10	11	12	13
1. EDEQ Restriction	1												
2. EDEQ Eating concern	0.67 **	1											
3. EDEQ Shape concern	0.67 **	0.78 **	1										
4. EDEQ Weight concern	0.63 **	0.74 **	0.85 **	1									
5. CIA-Personal	0.59 **	0.66 **	0.80 **	0.71 **	1								
6. CIA-Social	0.62 **	0.62 **	0.65 **	0.63 **	0.75 **	1							
7. CIA-Cognitive	0.45 **	0.61 **	0.61 **	0.54 **	0.67 **	0.67 **	1						
8. EDQOL Psychological	0.59 **	0.59 **	0.75 **	0.68 **	0.79 **	0.71 **	0.69 **	1					
9. EDQOL Physical/cognitive	0.51 **	0.62 **	0.63 **	0.55 **	0.63 **	0.71 **	0.82 **	0.71 **	1				
10. EDQOL Financial	−0.11	−0.06	0.10	0.04	0.09	−0.01	0.04 **	0.10	−0.00	1			
11. EDQOL Work/school	0.29 **	0.29 **	0.29 **	0.40 **	0.29 **	0.45 **	0.53 **	0.41 **	0.43 **	−0.09	1		
12. SF-12 Physical	−0.48 **	−0.37 **	−0.39 **	−0.32 **	−0.39 **	−0.42 **	−0.49 **	−0.49 **	−0.51 **	0.19	−0.34 **	1	
13. SF-12 Mental	−0.51 **	−0.56 **	−0.62 **	−0.62 **	−0.68 **	−0.72 **	−0.68 **	−0.65 **	−0.63 **	0.01	−0.46 **	0.51 **	1

Notes: ** *p* < 0.01. EDEQ (Eating Disorders Examination Questionnaire); CIA (Clinical Impairment Assessment); EDQOL (Eating Disorder-Specific Health-Related Quality of Life instrument); SF-12 (Short Form-12 Item Health Survey).

**Table 4:** Determinants of Health-Related Quality of Life.

Dependent Variables/ Predictors	*R_adj_* ^2^	*F*	β
EDQOL Psychological	0.611	19.846 ***	0.276 * 0.412 **
EDEQ Eating concern			
CIA Personal			
EDQOL Physical/cognitive	0.707	30.290 ***	0.290 ** 0.547 ***
CIA Social			
CIA Cognitive			
EDQOL Work/school/	0.317	6.648 ***	−0.439 ** 0.532 *** −0.394 *
EDEQ Eating concern			
CIA Cognitive			
CIA Personal			
SF-12 Physical/	0.314	6.545 ***	−0.385 ** −0.466 ***
EDEQ Restriction			
CIA Cognitive			
SF-12 Mental/	0.613	20.254 ***	−0.299 * −0.295 **
CIA Social			
CIA Cognitive			

Notes: * *p* < 0.05; ** *p* < 0.01; *** *p* < 0.001 EDE-Q (Eating Disorders Examination Questionnaire); CIA (Clinical Impairment Assessment); (EDQOL (Eating-Disorder Specific Health-Related Quality of Life instrument); SF-12 (Short Form-12 Item Health Survey).

## Data Availability

Data are unavailable due to ethical restrictions.
